# Egocentric Spatial Memory Deficit in Amnestic Mild Cognitive Impairment Revealed Through Virtual Reality: Cross-Sectional Study

**DOI:** 10.2196/79224

**Published:** 2026-02-25

**Authors:** Cosimo Tuena, Silvia Serino, Chiara Stramba-Badiale, Diana Biondi, Karine Marie Goulene, Marco Stramba-Badiale, Giuseppe Riva

**Affiliations:** 1 Department of Theoretical and Applied Sciences, eCampus University, Novedrate, Italy; 2 Applied Technology for Neuro-Psychology Lab, IRCCS Istituto Auxologico Italiano, 2 Alessandro Magnasco, Milan, 20145, Italy, 39 0261911 ext 2726; 3 Department of Psychology, Università degli Studi di Milano-Bicocca, Milan, Lombardy, Italy; 4 Department of Medicine, Neurology and Rehabilitation, IRCCS Istituto Auxologico Italiano, Milan, Italy; 5 Humane Technology Lab, Università Cattolica del Sacro Cuore, Milan, Italy

**Keywords:** spatial navigation, dementia, mild cognitive impairment, spatial cognition, virtual reality, digital health

## Abstract

**Background:**

Spatial navigation relies on egocentric and allocentric frames of reference, with the latter critically impaired in Alzheimer disease (AD) due to hippocampal involvement. Recent evidence suggests that egocentric representations may coexist within medial temporal lobe regions; however, their relative impairment in the early stages of AD remains unclear. Virtual reality offers a promising approach to bridge this gap by assessing spatial navigation abilities and providing sensitive digital biomarkers for AD.

**Objective:**

This study investigates spatial memory performance in individuals with amnestic mild cognitive impairment (aMCI) using a virtual reality object-location memory task that manipulated available cues during recall. An environmental boundary probed hippocampal-dependent allocentric processing, while a discrete landmark probed striatal-dependent egocentric strategies.

**Methods:**

Eighty participants (40 individuals with aMCI and 40 healthy controls [HC]) encoded object locations in a virtual arena and recalled them using either landmark or boundary cues.

**Results:**

Regardless of the spatial frame of reference, patients with aMCI demonstrated significantly poorer overall spatial memory than the HC group. Surprisingly, virtual egocentric error emerged as a predictor of aMCI diagnosis. No differences were observed in frame-switching, object-location binding, or across recall trials. Analysis of virtual encoding paths revealed that patients with aMCI exhibited centrifugal navigation away from the discrete landmark and toward the boundary of the arena. During recall, patients with aMCI demonstrated less effective usage of boundary and landmark cues than HC, as revealed by the distribution of response coordinates.

**Conclusions:**

These findings indicate that, in addition to well-documented allocentric deficits, egocentric spatial memory impairments provide a complementary and potentially sensitive marker within the AD continuum. This dual impairment of spatial cognition systems may enhance our understanding of navigational difficulties in aMCI and offers insight into the nature of spatial representations in aging.

## Introduction

Spatial navigation and memory represent cornerstone cognitive abilities that underpin our everyday functioning, playing a crucial role across the lifespan. These interconnected abilities enable self-orientation, object localization, wayfinding, and, in general, the maintenance of independence within one’s surroundings. Central to these abilities is how spatial information is represented. The environment can be represented using 2 different spatial frames of reference [1,2]. The egocentric frame uses a body-dependent representation (eg, “the chair is to my right”), while the allocentric frame uses a body-independent representation (eg, “the chair is close to the north-facing wall”). Crucially, the flexible switch and use of both spatial frames of reference sustain successful spatial navigation and memory [3-6].

Research has demonstrated that different types of cues in the environment tend to promote the use of different reference frames [7-9]. Specifically, extended environmental boundaries (such as walls) tend to promote allocentric processing, as they provide stable, viewpoint-independent reference frames. In contrast, discrete landmarks (such as a monument) often promote egocentric processing, as individuals tend to encode and recall target locations in relation to their own position relative to these salient features. Allocentric spatial memory relying on boundaries is mainly sustained by medial temporal regions, prevalently the right hippocampus and boundary cells in the subiculum and entorhinal cortex. In a virtual reality (VR) task, Lee et al [10] showed that encoding target locations close to the boundaries, compared to the center, elicited stronger theta oscillations than for target locations at the center of the environment, suggesting that the human subiculum is a key region associated with integrating boundary information into spatial memory, facilitating precise navigation and spatial recall. Conversely, egocentric spatial memory relying on landmarks is supported by the dorsal striatum and parietal cortex, with the medial prefrontal cortex serving as a critical hub for top-down modulation when both representations are similarly active [7,8,10-12].

As we age, the integrity of these spatial functions becomes increasingly significant, serving as a window into brain health. The literature indicates that spatial navigation and memory decline in normal aging, with older adults exhibiting increased use of egocentric navigation and diminished frame-switching abilities [13-15]. For instance, Schuck et al [16] manipulated geometric boundaries and local landmarks within a VR object-location (OL) memory task. Young adults demonstrated behavior patterns and medial temporal lobe activation consistent with hippocampal boundary-processing models. In contrast, older adults relied predominantly on landmark information, with their spatial learning correlating more strongly with caudate nucleus activity than hippocampal engagement, suggesting an age-related shift from a primarily hippocampal-dependent boundary-processing system to a caudate-mediated, landmark-oriented approach.

In pathological aging associated with Alzheimer disease (AD), this pattern progresses to a more severe impairment, with earlier and more marked deficits in allocentric navigation and eventual deterioration of compensatory egocentric strategies. Consistent with AD-specific neuropathological processes [17], significant allocentric deficits and frame-switching impairments have been consistently reported in AD dementia [18], even in the early stages [19]. Understanding when these deficits emerge is therefore critical for early detection. Mild cognitive impairment (MCI) has been proposed as a transitional stage from normal aging to dementia [20]. Specifically, individuals with objective impairment on memory tests, namely amnestic mild cognitive impairment (aMCI), have a heightened risk of developing AD compared to non-aMCI [21]. Consistent with this progression, individuals with aMCI not only report subjective navigational complaints but also manifest impairments in allocentric and egocentric spatial navigation, as well as in frame-switching abilities, especially from the allocentric to egocentric frame [22-25]. More recent research has begun to better reveal complex patterns of spatial impairment in this specific population. Using a VR boundary-based OL memory task, Castegnaro et al [26] found that individuals with aMCI recalled object locations less accurately than healthy controls (HC). They also exhibited increased object-binding errors, that is, difficulties in correctly associating each object with its original location, with objects frequently assigned to incorrect positions. Crucially, the distance between the recalled and encoded item locations achieved a good diagnostic classification performance (area under the curve [AUC]=0.89). However, aMCI with positive AD biomarkers performed similarly to individuals with negative AD biomarkers. The authors concluded that a possible egocentric strategy might have compensated for allocentric deficits in aMCI with AD. Nevertheless, regardless of the biomarker profile, participants with aMCI exhibited OL impairments compatible with entorhinal lesions.

Beyond allocentric deficits, there is some evidence for egocentric spatial impairments in aMCI. Studies using paradigms such as the human analog of the Morris water maze (MWM) [27] have found that individuals with aMCI plus multiple-domain impairments (aMCImd) and those with a hippocampal amnestic profile also manifested deficits in egocentric spatial memory [28,29]. In contrast, patients with focal impairment in memory (amnestic mild cognitive impairment, single-domain [aMCIsd]) were particularly impaired in allocentric spatial memory. Intriguingly, research has begun to characterize specific behavioral patterns during spatial navigation tasks. Individuals with aMCI showed responses less clustered around the target location during allocentric and egocentric conditions compared to HC and non-aMCI [27-29]. Behavioral markers during navigation tasks can reveal these underlying impairments.

Indeed, spatial behaviors tracked during a navigation task in response to specific environmental cues could express altered allocentric-hippocampal (eg, almost direct trajectory between starting and target location) and egocentric-nonhippocampal (eg, wall-hugging-centrophobic navigation, random exploration) strategies, which unveil impaired spatial navigation in AD [30]. Additionally, studies of AD risk carriers have identified distinctive navigational behaviors using VR tasks, including wall-hugging behavior when environmental boundaries were displayed and a preference for navigating closer to local landmarks to reach target locations when egocentric cues were presented [31-33].

In summary, while existing literature has demonstrated spatial navigation deficits in aMCI, significant questions remain regarding the precise nature of these impairments. Specifically, how patients with aMCI navigate when different types of spatial cues are available (environmental boundaries vs discrete landmarks) and the relative impairment in allocentric vs egocentric processing strategies remains poorly investigated.

To overcome these gaps, this study aims to exploit digital markers from the VR OL spatial memory task to evaluate boundary- and landmark-based recall strategies in aMCI compared to HC. Based on the existing literature, our study addresses two main objectives: (1) to evaluate spatial memory performance in aMCI compared to HC. Despite some evidence suggesting possible landmark-based (egocentric) impairments, we expect a focal allocentric spatial recall (boundary-based) deficit in aMCI, which we hypothesize will predict aMCI diagnosis; (2) to investigate navigational behavioral patterns. We expect patients with aMCI to display distinct navigation strategies compared to HC, specifically wall-hugging behavior in boundary-based conditions and centripetal navigation toward discrete landmarks in landmark-based conditions.

Finally, we explore these predictions in relation to aMCI subtypes, specifically aMCIsd and aMCImd variants.

## Methods

### Participants

Eighty older adults were recruited for this study and divided into 40 individuals meeting the core clinical criteria for MCI [34] and 40 HC. The MCI phenotype was established [34] in the patient group (aMCIsd=10 and aMCImd=30). The presence of cognitive impairment was determined by a team of neuropsychologists (CT, CSB, and DB) and a physician (KMG), based on the patient’s anamnesis, medical report, and neuropsychological diagnosis according to a screening battery. Objective cognitive impairment was determined by clinical judgment, including qualitative analysis of the participant’s test performance [35], and the psychometric equivalent scores method for Italian neuropsychological tests [36]. To reduce diagnostic bias, neuropsychological tests for each participant were evaluated by a team of neuropsychologists.

Inclusion criteria for the cognitively HC group were (1) age ≥65 years, (2) absence of cognitive impairment, (3) independence in functional abilities, and (4) absence of cognitive deterioration as determined by the Italian Mini-Mental State Examination (MMSE) cut-off [37].

MCI was diagnosed according to the core clinical criteria: (1) concern about a change in cognition expressed by the participant, an informant, or a clinician; (2) cognitive impairment in one or more domains; (3) independence in functional abilities; and (4) absence of a dementia diagnosis. Additional inclusion criteria were (1) age ≥65 years and (2) absence of cognitive deterioration as determined by the MMSE cut-off [37]. aMCIsd was determined when only the memory domain (verbal and/or visuospatial) was impaired on formal neuropsychological testing, whereas aMCImd was defined when, in addition to memory (verbal and/or visuospatial), participants had an objective impairment in an additional cognitive domain.

The following exclusion criteria were applied to both HC and MCI: (1) presence of an acute stroke or transient ischemic attack in the last 6 months, (2) diagnosis of neurological (eg, multiple sclerosis and Parkinson disease) and psychiatric disorders (eg, schizophrenia) or untreated psychiatric conditions (eg, mood and anxiety disorders), (3) history of traumatic brain injury with loss of consciousness, (4) physical or functional impairments (eg, musculoskeletal disorders and limb paresis/paresthesia) that could impair the use of the VR procedure, (5) severe or uncorrected visual impairment, and (6) presence of self-reported recurrent vertigo.

To compute the sample size, we used an effect size of *f*=0.32 [19]. For a mixed design (2 within-participant levels and 2 between-participant levels) with a power of 0.8 and an α of .05, the total sample size was 79 (calculated using the *pwr* R package).

Participants were recruited at the Outpatient Clinic of the Department of Medicine, Neurology, and Rehabilitation, IRCCS Istituto Auxologico Italiano—Mosè Bianchi, Milan. Table 1 reports the demographic and clinical baseline characteristics of the participants included in the analyses. An extended summary table is reported in Multimedia Appendix 1.

**Table 1.** Summary of participant characteristics (N=80).

Variable	aMCI^a^ (n=40)	HC^b^ (n=40)	*P* value
Age (years), mean (SD)	74 (6)	72 (6)	.30
Sex, n (%)			.10
Female	30 (75)	23 (58)	
Male	10 (25)	17 (42)	
Education, mean (SD)	12.3 (4.5)	13.9 (3.4)	.10
MMSE^c^ (points), mean (SD)	27.48 (2.10)	28.52 (1.88)	.02
FAB^d^ (points), mean (SD)	14.99 (2.17)	17.05 (1.12)	<.001
TMT-A^e^ (s), mean (SD)	36 (23)	28 (14)	.30
CF^f^ (points), mean (SD)	4.73 (1.59)	5.06 (1.00)	.50
DF^g^ (points), mean (SD)	5.69 (1.14)	6.26 (0.95)	.02
CSS^h^ (points), mean (SD)	12 (7)	19 (7)	<.001
PM-I (points), mean (SD)	4.17 (2.27)	5.82 (1.28)	<.001
PM-D^i^ (points), mean (SD)	4.11 (2.22)	5.84 (1.19)	<.001
GDS^j^ (points), mean (SD)	2.65 (2.62)	1.93 (2.12)	.20
ADL^k^ (points), mean (SD)	5.95 (0.22)	5.92 (0.27)	.70
IADL^l^ (points), mean (SD)	7.93 (0.47)	7.93 (0.27)	.30
VR^m^ encoding time (minute), mean (SD)	8.87 (5.14)	7.01 (3.73)	.07
PC^n^ expertise (points), mean (SD)	2.43 (1.19)	2.74 (1.27)	.27

a aMCI: amnestic mild cognitive impairment.

b HC: healthy control.

c MMSE: Mini-Mental State Examination.

d FAB: frontal assessment battery.

e TMT-A: trail-making test part A.

f CF: Corsi forward.

g DF: digit forward.

h CSS: Corsi supra-span learning.

i PM: prose memory immediate (I) and delayed (D).

j GDS: geriatric depression scale-short.

k ADL: activities of daily living.

l IADL: instrumental activities of daily living.

m VR: virtual reality.

n PC: personal computer.

### Materials

#### Neuropsychological Measures

The battery we administered comprised memory, language, attention, executive function, and visuospatial domains. The full set of neuropsychological tests and descriptions is reported in Multimedia Appendix 1.

#### OL Spatial Memory Task

We used a VR landmark- and boundary-based spatial memory recall similar to that described by Tuena et al [38]. Participants were seated in a comfortable position at a distance of 50 cm from the computer screen. In this task, a 3dRudder and a VR-ready computer (15.6-inch MSI GF63 Thin 11UC Intel Core i7-11800H, NVIDIA GeForce RTX 3050 4 GB, 16 GB RAM, and 512 GB SSD) were used to interact with the virtual environment. The 3dRudder is a circular platform designed for use while seated in a comfortable and safe position. To operate it, the user places the feet on top of the device, which is mounted on a semispherical base. This design enables intuitive movement control by tilting the feet in the desired direction or by pushing the toes or heels to move forward or backward. The device is equipped with inertial and pressure sensors that detect the user’s movements and convert them into virtual actions. The use of a motion foot pad instead of a classic joypad is intended to recruit lower-limb motor commands instead of hand and finger motor efferent copies, which is closer to real-world navigation [39].

The structure of the spatial memory task was as follows. During the encoding phase, participants were instructed to collect 4 items at a time and encode their locations within a circular arena (with a diameter of 50 virtual meters). Critically, during encoding, both the arena boundary (wall) and a fixed intra-arena landmark (obelisk; coordinates [−10.5, 10.5]) were simultaneously visible, allowing participants to encode object locations using both cues.

Items were presented randomly, and each object was collected 4 times in a random order. The 4 objects were the same for all the participants and were presented at the same fixed coordinates (cat [−5, −14], chair [−4, 20], bike [18, 10], and carrot [5, 17]). To visualize the object, participants had to navigate to its exact location. Upon reaching it, the object disappeared, and they proceeded to find the next one. The starting position (coordinates [0, 0]) of this phase was always the center of the circular arena, facing an arbitrary north.

During the immediate recall phase, participants had to remember and navigate to the exact location where the item (cued at the bottom of the screen) had previously been collected and then press a button on the keyboard to respond. The next object was then presented. Either the wall or the obelisk was randomly removed, forcing participants to rely primarily on one type of spatial cue. In the allocentric (wall) condition, the obelisk was removed, but the visible wall remained, encouraging boundary-based spatial memory. In the egocentric (obelisk) condition, the wall became invisible, but the obelisk remained visible, encouraging landmark-based spatial memory. Fixed distal cues (ie, mountain range and clouds) remained visible in both recall conditions as orientation cues. Each object was tested twice under both egocentric and allocentric conditions, resulting in a total of 16 trials (8 trials per recall condition).

**Figure 1.**
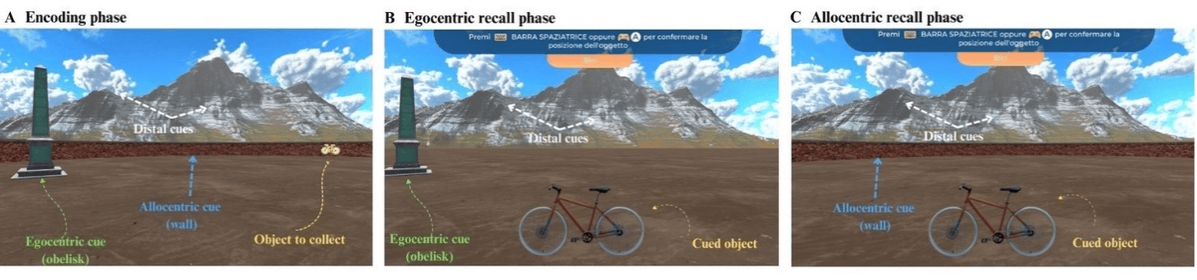
Object-location spatial memory task structure.In the encoding phase (A), participants were asked to learn the locations of 4 consecutive objects in a circular virtual arena containing both walls (allocentric cues) and a discrete landmark (egocentric cue). Participants collected each object 4 times in random order. During the recall phase (B and C), participants were cued to navigate to previously learned object locations; however, either the obelisk (B) or the wall (C) was randomly removed, forcing reliance on egocentric or allocentric spatial recall cues, respectively. While distal orientation cues remained visible in both conditions, each object was tested twice under both cue conditions (8 trials per spatial recall cue and 16 total trials).

An invisible wall was implemented as a constraint during the obelisk condition to ensure comparability between the 2 recall cue conditions. Figure 1 provides an overview of the task.

The recorded virtual variables were the encoding time to collect and encode the object locations; the Cartesian coordinates (x, y) of the recalled location; the Cartesian coordinates (x, y) of the paths made by participants during the encoding phase; and the error for each trial at recall, measured as the Euclidean distance (expressed in virtual Unity software meters) between the recalled position and the actual location. Details of the setup are shown in Figure S1 in Multimedia Appendix 1.

### Procedure

First, participants read and signed the consent forms to participate in the study. The study was conducted across 2 sessions. In the first session, participants were administered a comprehensive neuropsychological battery (refer to “Neuropsychological Measures” subheading). In the second session, participants completed the OL spatial memory task. Before the actual task, participants were instructed by the experimenter on how to use the 3dRudder in a training virtual environment provided by the 3dRudder software. Then, the encoding phase instructions were read: “Now, you will find yourself in a circular virtual arena where your task is to collect objects and memorize their locations, as you will be asked to recall them later. You will see one object at a time, and each object will appear four times in the same position to help you remember its location more easily. To aid in memorization, you can use the obelisk, the wall, the mountain range, and the fixed clouds as reference points. You can navigate within the arena using the 3dRudder. To collect an object, move directly over it. Once collected, it will disappear, and the next object will be presented.*”*

After this phase, the recall instructions were given: “You will be asked to place each object in the position where you originally collected it. However, either the obelisk or the wall will be randomly removed. Once you reach the location you believe is correct, press the spacebar on the keyboard to place the object and proceed to the next one. You will be asked to reposition each object multiple times, regardless of the accuracy of your previous answers.” Finally, a brief debriefing on the study’s aims was provided (the experimental sessions lasted approximately 1 hour and 15 minutes in total).

### Data Preparation and Statistical Analyses

R (version 3.6.3; R Foundation for Statistical Computing) software was used for the analyses. The main independent variables were group (HC vs aMCI) and spatial recall cue (egocentric vs allocentric). This resulted in a mixed between- and within-participants design.

Our study consisted of 2 sets of analyses corresponding to our 2 main research objectives: linear mixed-effects ANOVAs and generalized linear regression models (Objective 1), and binomial tests for density distributions (data points count) of Cartesian encoding and response coordinates (Objective 2).

The main dependent variable of interest for Objective 1 was the recall measure of Euclidean distance (error between encoded items’ Cartesian coordinates and recalled items’ Cartesian coordinates; hereafter referred to as error). In addition, to gain further insight into spatial memory performance, we assessed OL binding errors and frame-switching cost. A thorough description of the statistical methods for Objective 1 is reported in Multimedia Appendix 1. To reduce bias, we also controlled for computer expertise (refer to Table 1 and Multimedia Appendix 1 for details) in the groups and carried out analyses adjusted for potential confounding variables (eg, age, gender, and global cognition). Objective 2 details are reported in the “Data Preparation and Statistical Analyses for Navigational Behavioral Patterns” section. The significance level was set to .05 in all analyses.

#### Data Preparation and Statistical Analyses for Navigational Behavioral Patterns

First, we analyzed path length in the HC and aMCI groups at encoding. To quantify participants’ movement within the arena, we computed the Euclidean distance between consecutive position samples (path Cartesian coordinates) for each participant. For each participant, we then computed the mean step distance (average distance traveled per time step) and the total distance (sum of all stepwise distances). Group differences in mean step distance between participants with HC and aMCI were assessed using an independent samples *t* test (with Welch correction for unequal variances). The same procedure was adopted for paths at recall. Encoding and recall path coordinates were sampled at 1 Hz (2/80 files could not be saved due to technical issues).

We also computed the Euclidean distance of each path point at encoding from the fixed landmark and the center of the arena and averaged this distance for each participant to carry out an independent samples *t* test (Welch correction for unequal variances) between the 2 groups.

Second, to analyze the spatial distribution of encoding and response patterns in the arena, we developed a systematic approach that allowed us to examine potential biases toward specific reference points in both egocentric and allocentric conditions, similar to that described by Lee et al [10]. Recall coordinates corresponded to each participant’s response.

Regarding the allocentric condition data point densities (encoding paths and recall data points), we divided the circular arena (radius=25 units) into inner (radius≤12.5 units) and outer annular regions to examine biases toward the center or boundary. We calculated point densities in each region by dividing point counts by their respective areas (inner area=π x 12.5² and annular area=π x [25² – 12.5²]). To determine whether participants showed significant deviations from the expected distribution based on relative areas (25% inner and 75% outer), we conducted binomial tests comparing observed proportions to the expected proportions. Additionally, we calculated density ratios (inner to outer) to quantify preferences toward central or peripheral regions.

For the egocentric condition densities (encoding paths and recall data points), we compared the quadrant containing the egocentric (obelisk) landmark (coordinates [−10.5, 10.5]) with the diametrically opposite quadrant. Specifically, we assessed whether participants placed objects more frequently in the upper-left quadrant (containing the obelisk) compared to the lower-right quadrant. We performed binomial tests to determine whether the proportion of points in the landmark quadrant significantly differed from 0.5 (the expected proportion under the null hypothesis of equal distribution between the 2 quadrants). We also calculated density ratios between these quadrants to quantify the strength of any spatial recall cue–based bias. In addition, we also calculated the ratio of density ratios between groups to quantify the relative strength of spatial biases across clinical populations.

Finally, before proceeding to compute data point densities for recall, we ensured that responses were unbiased to fixed object locations. We computed an intra-arena landmark attractor index, normalized by the distance of each object from the landmark. This index quantifies how much a response is shifted toward the intra-arena landmark ([d_target_ – d_responseLM_]/d_target_), where d_target_ is the Euclidean distance from the object’s original location to the landmark and d_responseLM_ is the distance from the participant’s response to the landmark. Positive values indicate a bias toward the landmark, whereas negative values indicate a bias toward the object’s true location. We also computed an arena center attractor index, normalized by the distance of each object from the arena center. This index quantifies how much a response is shifted toward the center of the arena ([d_target_ – d_responseC_]/d_target_), where d_target_ is the distance from the object’s original location to the arena center, and d_responseC_ is the distance from the response to the center. Negative values indicate a bias toward the center of the arena, whereas positive values indicate a bias toward the object’s true location. These 2 attractor indices were analyzed separately with a linear mixed-effects model, with participants and objects as random effects and group (HC vs aMCI) as a fixed predictor.

### Ethical Considerations

The study was conducted in accordance with the Declaration of Helsinki and approved by the Ethics Committee of Istituto Auxologico Italiano (approval no 2023_01_31_11). Written informed consent was obtained from all participants before their participation in the study. All data were de-identified prior to analysis. Participants were assigned a unique alphanumeric code, and any directly identifying information was removed and stored separately in compliance with ethical and data protection regulations.

## Results

### Overview

Table 1 reports the key characteristics of the 2 groups. The extended table with all the neuropsychological measures and multiple Pearson correlations in the whole sample between spatial error performance and those measures is reported in Tables S1 and S2 in Multimedia Appendix 1.

### Objective 1

#### Differences Between aMCI and HC and Spatial Memory Recall Cues

To study group and spatial recall cue differences, we performed a linear mixed-effects ANOVA for the error metric.

**Figure 2.**
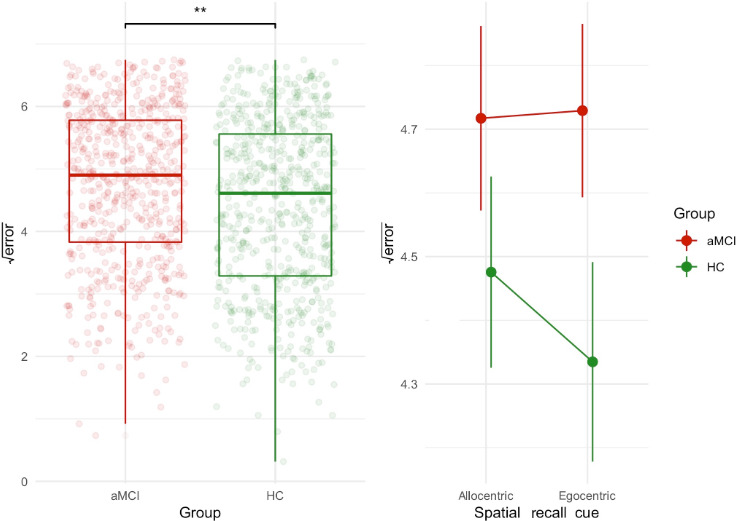
Group differences in the spatial memory task. aMCI: amnestic mild cognitive impairment; HC: healthy controls. **P<.01

We found a significant main effect of the group variable (*F*_1,77_=8.37; *P*=.005; and *f*=0.33). Regardless of the spatial recall cue, patients with aMCI demonstrated a higher mean error of 23.9 (SD 11.3) virtual meters than HC, who showed a mean error of 21.3 (SD 11.7). We did not find a main effect of spatial recall cue (*P*=.37) or the interaction between spatial recall cue and group (*P*=.27). The significant effect of the group variable (*P*=.03) was also retained after adjusting for the covariates [26], namely age, years of education, gender (*P*=.03), retrieval time for each object, global cognition, age by time, and age by gender (*P*=.02). Figure 2 shows the results of these analyses with (A) a significant main effect of the group variable on spatial memory error, and (B) nonsignificant spatial memory performance by spatial recall cue in the groups (mean and 95% CIs), with a detailed summary of these analyses reported in Multimedia Appendix 1.

Crucially, an ANOVA was conducted to explore any differences in recall time. We did not find a main effect of group (*P*=.07), spatial recall cue (*P*=.92), nor the interaction between group and spatial recall cue (*P*=.65). Results were unchanged when the model was adjusted for age, years of education, gender, and global cognition.

We also performed a polynomial linear mixed-effects model to investigate the performance across testing trials.

Concerning the error, the results of the group main effect above were replicated (*P*=.005). Crucially, we did not find any improvement or deterioration across testing trials (*P*=.40). The main effect of spatial recall cue (*P*=.37), the interaction between group and spatial recall cue (*P*=.27), the interaction between trials and group (*P*=.43), between trials and spatial recall cue (*P*=.98), and among trials, spatial recall cue, and group (*P*=.37) were not significant.

#### OL Memory Binding in aMCI and HC and Spatial Memory Recall Cues

To analyze the effects of group and spatial recall cues on binding errors, we performed a Poisson regression. Neither the main effect of group (*P*=.24) nor spatial recall cue type (*P*=.85) reached statistical significance. The interaction between group and spatial recall cue type was also nonsignificant (*P*=.51). Results remained unchanged after adjusting for the covariates suggested by Castegnaro et al [26], namely age, education, gender, MMSE, and gender by age. Refer to Figure S2 for the binding errors in the groups. Figure S2 in Multimedia Appendix 1 shows the binding errors for the 4 objects.

#### Frame-Switching Cost in aMCI and HC

To analyze the effects of group (aMCI vs HC) and recall cue switching condition (ego-to-allo vs allo-to-ego vs no switch) on error, we performed a linear mixed-effects ANOVA.

We found a significant main effect of group (*F*_1,78_=4.71; *P*=.03; and *f*=0.25). Regardless of the switching condition, HC had higher retrieval accuracy (mean 21.3, SD 6.89) compared to the aMCI group (mean 23.5, SD 6.89). The switching condition (*P*=.97) and the group-by-switching condition (*P*=.18) were not significant. We also performed a model adjusting for age, education, gender, retrieval time, MMSE, age by gender, and age by time; however, in this case, the main effect of group did not survive (*P*=.11). No other significant effects were found for the adjusted model. See Figure S3 in Multimedia Appendix 1 shows the unadjusted frame-switching cost results.

In addition, we performed a logistic regression analysis to explore the predictive power of the recall cue switching condition in classifying aMCI vs HC. We found that only the no-switch error (*β*=.12; *P*=.01) was positively associated with aMCI diagnosis (OR 1.13, 95% CI 1.03-1.25). The other switch predictors were not significant (ego-to-allo: *P*=.78 and allo-to-ego: *P*=.57). The association for no-switch error remained significant after adding relevant covariates (*P*=.04), such as age, education, gender, MMSE (*β*=−.4; *P*=.01), and age by gender. This suggests that frame switching is impaired in both conditions, as the no-switch condition was a good predictor of aMCI, whereas the other 2 conditions were not.

#### aMCI and HC Classification and Spatial Memory Recall Cues Performance

We investigated the predictive power of group classification of egocentric and allocentric performances using a logistic regression model.

Regarding the error, our model revealed that egocentric error was a significant predictor of group status (*β*=.98; *P*=.01), with higher error associated with aMCI diagnosis. In contrast, allocentric error did not significantly predict group membership (*β*=.33; *P*=.39). The intercept of the logistic regression was significant (*β*=−5.96; *P*=.01); when egocentric and allocentric errors were 0, there was a greater chance of being classified as HC.

The egocentric error effect also remained significant (*β*=1.11; *P*=.03) after controlling for relevant covariates, namely age, education, gender (β_male_=−1.65; *P*=.02), retrieval time, and MMSE (*β*=−.43; *P*=.01) [26]. This pattern of results suggests a selective impairment in egocentric spatial navigation in patients with aMCI compared with HCs, while allocentric spatial representations appear relatively preserved. Table 2 reports the full model results.

**Table 2.** Full model logistic regression results. Reference class: aMCI^a^.

Predictor	OR^b^ (95% CI)	*P* value
Egocentric error (vm)^c^	3.02 (1.20-8.96)	.03
Allocentric error (vm)	1.25 (0.51-3.15)	.60
Age (years)	1.00 (0.91-1.09)	.90
Education (years)	0.93 (0.81-1.07)	.30
Sex		
Male	0.19 (0.04-0.71)	.02
MMSE^d^ (points)	0.65 (0.46-0.88)	.008
Retrieval time (s)	0.99 (0.95-1.02)	.50

a aMCI: amnestic mild cognitive impairment.

b OR: odds ratio.

c vm: virtual meters.

d MMSE: Mini-Mental State Examination.

**Figure 3.**
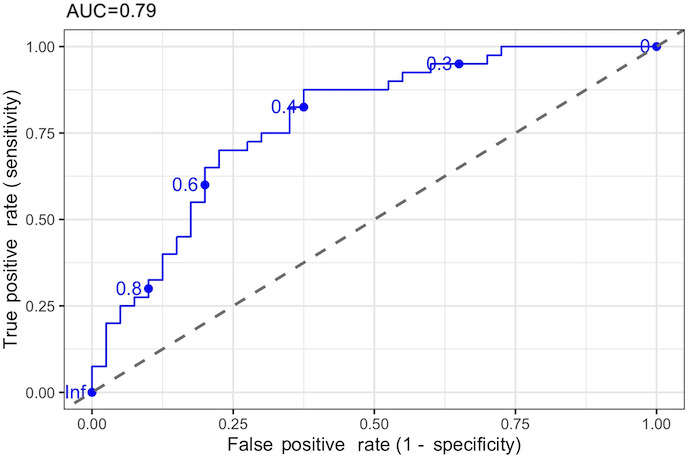
Receiver operating characteristic (ROC) curve of the full logistic regression model. AUC: area under the curve.

A receiver operating characteristic curve analysis was conducted to evaluate the diagnostic performance of the full model for the classification of aMCI. The AUC was 0.79, indicating moderate discriminatory ability (Figure 3). The optimal threshold was determined using the Youden index, which maximizes the sum of sensitivity and specificity. The analysis yielded an optimal probability cutoff value of 0.43. At this threshold, the test demonstrated a sensitivity of 0.88 and a specificity of 0.63, with a global accuracy of 0.75.

We also analyzed the cognitive measure of the model separately outside the logistic regression model. A receiver operating characteristic curve analysis was conducted to evaluate the diagnostic performance of the egocentric error measure. The AUC was 0.70, indicating moderate discriminatory ability. The optimal threshold was determined using the Youden index, which maximizes the sum of sensitivity and specificity. The analysis yielded an optimal cutoff value of 4.35. At this threshold, the test demonstrated a sensitivity of 0.80 and a specificity of 0.58. This means that egocentric error is particularly effective for ruling out aMCI, given the high sensitivity and, consequently, the low number of false negatives (ie, when egocentric error is below the cutoff, the participant is likely to be HC). Conversely, values above the cutoff may result in false-positive classifications because of the lower specificity.

Finally, MMSE showed an AUC of 0.68, indicating poor discriminatory ability. The analysis yielded an optimal cutoff value of 28.15. At this threshold, the test demonstrated a sensitivity of 0.68 and a specificity of 0.65. Consequently, the egocentric error is more accurate than MMSE for classifying healthy older adults.

Despite not being a significant predictor, we also computed a receiver operating characteristic curve analysis for the allocentric error. The AUC was 0.61, and the best cutoff was 4.87, which yielded a sensitivity of 0.5 and specificity of 0.75. This is specific to the egocentric error measure; in this case, the higher specificity is a better predictor of aMCI given the lower number of false positives, and a positive score above the cutoff may indicate aMCI.

### Objective 2

#### Encoding Paths in aMCI and HC

**Figure 4.**
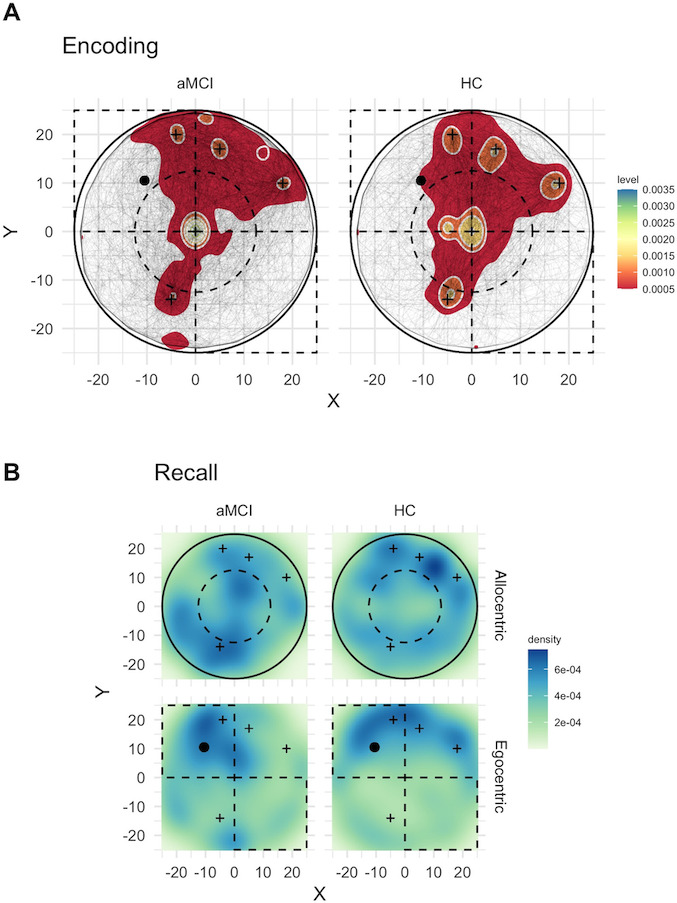
Density distributions within the virtual environment. aMCI: amnestic mild cognitive impairment; HC: healthy control.

We computed the patterns of encoding Cartesian (path) coordinates to explore navigation behavior in this phase. This data type allows us to explore the paths within the virtual environment during the encoding phase of the object locations. Refer to Figure 4 for the regions used in these analyses and the path density distributions in the groups at encoding. In the figure, (A) shows heat maps representing the density of encoding path points, with dark blue indicating higher concentration. Black lines represent paths taken by participants during the encoding phase, and black cross markers show object positions. The black dot represents the obelisk landmark (–10.5, 10.5), with dashed lines delineating the analyzed upper-left and lower-right quadrants; the solid circle represents the arena boundary (radius=25 units), with a dashed inner circle (radius=12.5 units) separating central and annular regions. (B) Shows heat maps of response point densities at recall, with dark blue indicating higher concentration. Black cross markers show encoding object positions. In the egocentric condition, the black dot represents the obelisk landmark (–10.5, 10.5), and in the allocentric condition, the solid circle represents the arena boundary (radius=25 units), with a dashed inner circle (radius=12.5 units) separating central and annular regions. Both cues were presented during encoding. Allocentric refers to a boundary cue presented, but no local landmark, and egocentric refers to local landmark presented, but no boundary. Legends show density levels.

First, we calculated whether the groups differed in terms of path length. A *t* test showed that groups did not differ in path length (*P*=.08). Along with encoding time (refer to Table 1), this result shows that time and path do not differ quantitatively in aMCI from normal cognitive aging.

Second, a Welch 2-sample *t* test was conducted to compare the mean distance from the intra-arena landmark between participants with aMCI and HC. There was a significant difference in mean distance between the 2 groups (*t*_63_=2.35; *P*=.02). Participants with aMCI (mean 19.57, SD 1.34) maintained a greater average distance from the landmark during encoding navigation compared to HC participants (mean 18.65, SD 2.05). Similarly, a Welch 2-sample *t* test was conducted to compare the mean distance from the center of the arena between participants with aMCI and HC. There was a significant difference in mean distance between the 2 groups (*t*_76_=2.35; *P*=.02). Participants with aMCI (mean 15.45, SD 1.96) maintained a greater average distance from the center compared to HC participants (mean 14.45, SD 1.77). These analyses showed a tendency to navigate closer to the boundary of the arena and away from the landmark, which is replicated in the data point density analyses.

HC demonstrated significantly higher point density in the inner circle compared to patients with aMCI (36.4% vs 29.16%, binomial test *P*<.001), suggesting a centripetal pattern during the encoding phase for HC. Conversely, patients with aMCI showed significantly higher concentration in the outer ring (70.83% vs 63.6%, binomial test *P*<.001), suggesting a centrifugal bias toward the boundary during the encoding phase.

Quadrant analysis revealed that HC had significantly higher point density in the top-left quadrant (region that contained the egocentric cue [obelisk]) compared to participants with aMCI (27.75% vs 24.37%; *P*<.001), suggesting a bias toward the egocentric landmark for HC. Conversely, participants with aMCI showed a significantly higher concentration in the bottom-right quadrant (14.98% vs 13.64%; *P*<.001), suggesting a centrifugal pattern away from the egocentric cue.

Overall, the results indicate a centrifugal pattern of encoding navigation for aMCI away from the egocentric cue and toward the boundary (away from the center of the arena) and a more clustered pattern for HC toward the center of the arena and the egocentric cue.

#### Point Pattern Analysis and Spatial Recall Cue in HC and aMCI

We analyzed the response patterns to investigate behavioral recall differences between the groups.

First, we calculated whether the groups differed in terms of path length. Results showed that aMCI had significantly (*t*_74_=3.64; *P*<.001) longer paths (mean 2.83, SD 0.89) compared to HC (mean 2.16, SD 0.74).

Second, normalized attractor index analyses for egocentric and allocentric performance showed that the HC group had a lower bias toward the landmark (*β*=−.21; SE=0.07; *P*=.007) and toward the center of the arena (*β*=−.14; SE=0.06; *P*=.02) compared to the aMCI group. This ensured that the following analyses were relatively unbiased by fixed object locations. Indeed, this result was replicated in the data point densities analyses reported in the next paragraph: aMCI recalled locations in the allocentric condition toward the center of the arena and toward the landmark during the egocentric condition.

Regarding data point densities, we started by investigating the point pattern for allocentric performance. We analyzed the spatial distribution of response points in the 2 groups by examining the density patterns relative to the structure of the circular arena. For this purpose, we divided the circular arena into an inner region (within the inner circle of radius 12.5 units, far from the wall) and an outer annular region (between the inner and outer circles, 12.5< radius ≤25 units, closer to the wall). The inner area constituted 25% of the total area, while the outer area represented 75%.

Refer to Figure 4 for the regions used in these analyses, the groups’ density point distributions, and recall cue conditions.

The HC group placed 17.81% of their recall points in the inner area (57/320 points) and 82.19% in the outer area (263/320 points). This distribution significantly differed from the expected proportions based on the inner and the outer portion of the arena (binomial test; *P*=.002).

In contrast, the aMCI group placed 23.75% (76/320) of their recall points in the inner area and 76.25% (244/320) in the outer area. This distribution did not significantly differ from the expected proportions for the inner and outer sections of the arena (binomial test; *P*=.65). To account for the different sizes of the inner and outer areas, we calculated point densities (points per square unit). In the HC group, the density was 0.11 points/unit^2^ in the inner area and 0.18 points/unit^2^ in the outer area, yielding an inner-to-outer density ratio of 0.65.

For the aMCI group, the density (points per unit area) was 0.15 points/unit^2^ in the inner area and 0.17 points/unit^2^ in the outer area, resulting in an inner-to-outer density ratio of 0.93. The difference in density ratios between groups was −0.28, indicating that patients with aMCI showed a more balanced distribution of points between inner and outer areas than HC participants, who demonstrated a stronger preference for the outer area. This suggests a potential alteration in allocentric spatial memory strategies in aMCI, characterized by reduced usage of the circular boundary as a spatial reference point compared with healthy older adults.

We then inspected the egocentric performance. We examined the spatial distribution of response points across opposing arena quadrants in patients with aMCI and HC. We focused on comparing the upper left quadrant, which contained the egocentric cue (obelisk at coordinates [−10.5, 10.5]), with the diametrically opposite lower right quadrant.

In the HC group, 32.19% (103/320) of recall points were positioned in the upper left quadrant, while 17.19% (55/320) were located in the lower right quadrant. This asymmetric distribution between the 2 quadrants was statistically significant (binomial test; *P*<.001).

Similarly, the aMCI group placed 32.50% (104/320) of recall points in the upper left quadrant and 20.00% (64/320) in the lower right quadrant. This distribution also showed a significant preference for the upper left quadrant (binomial test; *P*=.003).

To account for the spatial area, point densities were calculated for each quadrant. In the HC group, the point density was 0.21 points/unit^2^ in the upper left quadrant and 0.11 points/unit^2^ in the lower right quadrant, yielding an upper-left–to–lower-right density ratio of 1.87.

For the aMCI group, the point density was 0.21 points/unit^2^ in the upper left quadrant and 0.13 points/unit² in the lower right quadrant, resulting in a density ratio of 1.62. The difference in density ratios between groups was 0.25, indicating that HC participants showed a slightly stronger preference for the upper left quadrant compared to patients with aMCI. The absolute differences in point densities between groups were minimal in both the upper left (−0.01 points/unit^2^) and lower right (−0.01 points/unit^2^) quadrants, with patients with aMCI showing slightly higher densities in both regions.

### Explorative Analyses in aMCIsd and aMCImd

We used robust methods to compare encoding time and spatial error differences, and robust logistic regression between aMCI phenotypes, given unequal group sizes. A comprehensive description of aMCI phenotype differences is reported in Multimedia Appendix 1. To summarize, participants with aMCIsd spent significantly more time (*P*=.004) in the encoding phase compared to participants in the aMCImd group. Egocentric and allocentric errors could not predict aMCIsd or aMCImd diagnosis. Regarding spatial behavior during encoding, aMCIsd showed a statistically significant centrifugal (*P*<.001) pattern away from the local landmark, whereas aMCImd navigated closer to the obelisk; no differences were observed between the central and annular regions of the arena. Regarding spatial behavior during recall, no differences were reported again during the boundary-recall condition between the 2 groups in the central and annular regions. The aMCImd group showed a significantly higher response density closer to the discrete landmark compared to the control region (*P*=.008); this pattern was not observed in aMCIsd.

## Discussion

### Principal Findings

In this study, we sought to profile aMCI spatial memory performance using a VR OL task that manipulated available spatial cues during recall: environmental boundaries (engaging allocentric processing) vs discrete landmarks (engaging egocentric processing). Participants were asked to collect objects and encode their locations in a virtual arena with discrete landmarks and boundaries. At retrieval, they were asked to put objects in place where they were previously collected, either using the discrete landmark or the boundary. We expected patients with aMCI to show impaired spatial memory, particularly in boundary-based recall conditions, and to exhibit distinct navigational strategies, including wall-hugging behavior and preferential use of local landmarks.

Our predictions were only partially confirmed by the study results. Concerning the first objective, we found that regardless of the spatial recall cue, patients with aMCI exhibited lower spatial memory performance than HCs; surprisingly, the egocentric error, rather than the allocentric error, was a significant predictor of aMCI diagnosis. No differences were found between aMCIsd and aMCImd. Second, we did not find deterioration in testing trials or any OL memory binding deficits in aMCI. Finally, contrary to the previous literature, we found no impairment in switching abilities (from egocentric to allocentric and from allocentric to egocentric).

Concerning our second research objective, we found a wall-hugging–like behavior in aMCI and navigation away from the discrete landmark (especially in aMCIsd) during the encoding phase.

Interestingly, patients with aMCI showed no quantitative encoding deficits (ie, comparable encoding time and path length to HCs), but differed qualitatively in their encoding strategies. Regarding the recall phase, patients with aMCI gathered the responses at the center of the environment (in both phenotypes) in the allocentric condition and toward the landmark (especially in aMCImd) during the egocentric recall cue condition. Quantitatively, the 2 groups differed in path length but not in retrieval time.

### Objective 1

While Castegnaro et al [26] showed that boundary-based recall has promising discriminative performance between aMCI and HC groups, intriguingly, we also found that higher egocentric error was associated with aMCI diagnosis. First, this is in line with a recent systematic review [22] that showed both egocentric and allocentric deficits in aMCI in real-world and VR tasks, extending the traditional focus on allocentric impairments [26-29,40], including egocentric impairments across the AD continuum. Our findings are consistent with the well-established association between AD and allocentric spatial memory deficits, yet they also highlight a complementary impairment emerging along the AD continuum, especially in its early stages. Although allocentric performance did not reach significance in the logistic model, it proved useful for ruling in the condition, whereas egocentric error was more informative for ruling out an aMCI diagnosis. This pattern aligns with a recent diagnostic meta-analysis conducted by our group [41], which showed that both egocentric and allocentric measures contribute valuable diagnostic information for AD, supporting a complementary, rather than competing, approach.

Our findings on egocentric impairment also align with recent results [42-45] that have provided compelling evidence for the coexistence of boundary egocentric vectors in medial temporal structures with allocentric representations. These body-based representations can integrate head direction signals to form a map of allocentric space, aiding navigation toward significant locations within an environment. Despite the right hemisphere, and particularly the hippocampal region, being the dedicated substrate for allocentric spatial computations [7,46], the human left hippocampus supports landmark-based spatial memory by sequencing spatiotemporal navigation (eg, the sequence of body-based movements at a given location and time) in an episodic-like format [11,46]. Aligned with this, patients with aMCI with hippocampal memory deficit, compared to those with frontal memory deficit, showed a greater deficit in egocentric spatial memory [28,29]. In addition, a critical diagnostic consideration is that current MCI neuropsychological classifications do not consider the modality of memory impairment (eg, verbal vs visuospatial aMCI), potentially masking important cognitive differences that affect spatial navigation abilities. Given the heterogeneity of MCI, caution should be adopted when designing spatial navigation and memory tasks and interpreting their results. Finally, our results confirmed that egocentric spatial memory is preserved in healthy aging and is a good metric to distinguish between aMCI and HC groups; this aligns in particular with a previous study that showed preserved landmark-based compared to boundary-based spatial memory in aging [16]. It is also worth noting that, although gender showed a significant effect as a covariate in both the ANOVAs and the logistic regression, the main results remained significant. This suggests that our findings are not biased by participants’ sex. Nevertheless, future research should further investigate potential gender differences in aMCI to better elucidate possible distinctions between egocentric and allocentric processes.

Our study revealed no performance improvement or deterioration in either the aMCI or HC groups. This contradicts previous research suggesting consolidation deficits in aMCI [27-29]. The absence of deterioration, paired with similar encoding times across groups, may indicate frontal involvement in retrieval strategies rather than hippocampal consolidation deficits. This aligns with evidence highlighting dysexecutive memory impairments in aging, aMCI, and AD, as well as the critical role of executive functions in supporting spatial navigation [47-49].

Unlike findings from a previous study [26] showing performance differences during allocentric recall, we found similar OL binding performance across cue types. Our stricter age criterion (≥65 years) likely explains why aMCI and HC performed comparably, as normal aging involves associative memory binding deficits linked to hippocampal and frontal dysfunction [49-51]. However, unimpaired OL binding was also found in AD genetic risk carriers [52].

Finally, while HC demonstrated lower switching costs than aMCI overall, we observed comparable frame-switching costs between groups, contradicting previous findings [24] of differential impairments in allocentric-to-egocentric vs egocentric-to-allocentric switching. This suggests that shifting between spatial representations represents a fundamental process affected by both normal aging and AD [5,15,18,53].

### Objective 2

Concerning our second objective, we confirmed that the pattern of encoding paths and responses during retrieval could be a cognitive marker of aMCI [27-29]. However, we extended previous research by demonstrating specific biases in virtual navigation according to spatial recall cues and group.

Concerning the encoding paths, we found that individuals with aMCI, at the group level, tended to explore the arena closer to the boundaries, avoiding the center of the environment. In contrast, the inverse pattern was found in HC. A similar centripetal pattern was found for the discrete landmark in aMCI, while HC tended to navigate closer to the landmark. The former finding is compatible with a thigmotaxis-like (wall-hugging) navigation behavior observed in animals in the MWM [30]. This spatial behavior has been observed in several species while exploring a novel environment and is considered a safety-seeking strategy in the early phases [54]. However, thigmotaxis could also reflect pathological spatial behavior. Indeed, rats with lesions to the hippocampus exhibit perseverative thigmotaxis and an inability to learn more successful strategies [55]. Similarly, lesions to the dorsomedial striatum lead to increased thigmotaxis due to the inability to switch from this strategy to a more efficient one [56]. Importantly, thigmotaxis has been observed in humans as an early strategy in novel environments and is associated with higher levels of fear and avoidance bias toward fear-mobilizing situations or agoraphobia [57,58]. Extensive or pathological use of egocentric behavior, such as thigmotaxis, hampers the capacity to switch between spatial navigation strategies, which is crucial to forming a gestalt-like representation of the space, also known as the cognitive map [57,59]. In line with this, research on path integration (ie, the ability to track one’s current position using current self-motion cues) has provided compelling evidence that environmental cues, particularly boundaries and, to a lesser extent, discrete landmarks, can correct navigation errors [8,60,61]. The boundary-hugging bias might reflect a compensatory egocentric mechanism, possibly also driven by spatial anxiety due to disorientation, that leads individuals with aMCI to walk closer to the boundaries virtually. Complementary to this, the local landmark is not perceived as a useful affordance [39] for navigation purposes in the aMCI group. This latter pattern is particularly true for aMCIsd, which also showed longer encoding times than aMCImd, suggesting a specific deficit during the learning process. The landmark-based deficit is compatible with findings on the human analog of the MWM in the egocentric recall test [28] and path integration movements toward the landmark in individuals with genetic risk for AD [33]. In contrast, HC had higher path density in the center of the arena, hinting at an allocentric-hippocampal strategy between object locations, akin to the “direct path” observed in MWM [30]. Collectively, the results show that in addition to an allocentric navigation deficit, aMCI, especially in aMCIsd, is accompanied by an egocentric navigation deficit at encoding.

Concerning the recalled Cartesian coordinates, we found that HC effectively leverages environmental boundaries for spatial memory recall; this boundary-anchored strategy may be partially compromised in older people with aMCI, who tended to remember object locations at the center of the arena. Indeed, the human hippocampus contains boundary cells that help to encode item locations with respect to distances from a boundary and support effective recall [10]. Conversely, patients with aMCI (especially aMCImd) and HC effectively used the egocentric reference point (obelisk) when performing spatial memory recall, with a strong preference for placing objects in the quadrant containing this landmark, especially for the HC. The results were similar in both aMCI phenotypes. This is partially in line with path integration based on boundary and landmark cues in individuals at risk for AD [33]. Bierbrauer et al [33] found that the distance of the landmark to the target condition is critical for recall performance during the landmark cue condition. At the same time, for young adults with genetic risk, this is true for the boundary cue. In addition, they showed that AD risk carriers walked toward the landmark while on the path toward the target location.

However, this finding must be interpreted considering the encoding path patterns, error performance, and group discrimination. In the former case, we demonstrated that participants with aMCI during the encoding phase tended to navigate closer to the boundary. At the same time, at recall, they remembered object locations in the center of the arena. The opposite pattern was found for HC. This hints at an allocentric spatial memory impairment that, however, is not particularly discriminative between the 2 groups.

In the latter case, the bias toward the landmark at recall, in the absence of an environmental boundary, is indicative of defective egocentric recall performance, which arises as a first step in the encoding phase, possibly due to a primary bias toward the boundary in aMCI, leading participants to navigate away from the local landmark. This behavior was not observed in HC. Consequently, while the saliency of the landmark with respect to object locations is partially preserved during the testing phase, the recall accuracy for the actual location is impaired, allowing for better discrimination between groups. Finally, both MCI phenotypes have, in addition to an allocentric, a different egocentric deficit: aMCIsd does not use the egocentric cue as an affordance at encoding, while aMCImd does at recall.

Crucially, we found that qualitative measures of navigation during encoding revealed specific deficits that were not detected by quantitative measures such as total time spent in the environment (encoding time) or path length. This underscores the importance of analyzing behavioral strategies, which may capture the distinctive spatial navigation deficits observed in aMCI and potentially in AD. Similarly, although recall time did not differ between the groups, the combination of path length and qualitative patterns of spatial memory responses indicates that participants with aMCI exhibit both quantitative and qualitative impairments in spatial navigation. This aligns with the long, and sometimes overlooked, tradition of qualitative analysis in the neuropsychology of memory [35]. Quantitative scores alone provide limited information; they should always be complemented by an examination of how patients perform a given task. Previous research in spatial navigation has demonstrated that response patterns reveal underlying spatial memory behaviors, providing valuable insights into the deficits observed in aMCI [27-29].

### Limitations

Our study has some limitations that should be acknowledged. First, we lack AD biomarkers that could help to define the underlying pathology better, and we collected participants in only 1 center. Second, we used a nonimmersive VR setup, which lacks crucial bodily (eg, vestibular) information, and this could have affected spatial memory results. Future studies could overcome these limitations by using an immersive apparatus equipped with an eye-tracking system to control for visual exploration, thereby discovering innovative digital biomarkers for aMCI diagnosis in larger cohorts.

### Conclusion

To conclude, our findings suggest that suspected AD pathology affects not only allocentric spatial memory in aMCI but also impairs egocentric spatial representations. The latter constitutes a complementary critical process in navigation and could represent a useful digital biomarker in the progression of AD. This dual impairment of spatial cognition provides a more comprehensive understanding of the navigational difficulties in aMCI and may offer new avenues for detection and intervention with digital health technologies.

## Supplementary material

10.2196/79224 Multimedia Appendix 1 Neuropsychological measures and spatial memory tasks details and analyses.

## Abbreviations

AD: Alzheimer disease
aMCI: amnestic mild cognitive impairment
aMCImd: amnestic mild cognitive impairment, multiple-domain
aMCIsd: amnestic mild cognitive impairment, single-domain
AUC: area under the curve
HC: healthy control
MCI: mild cognitive impairment
MMSE: Mini-Mental Score Examination
MWM: Morris water maze
OL: object-location
VR: virtual reality
