# Supplementary material for: Egocentric Spatial Memory Deficit in Amnestic Mild Cognitive Impairment Revealed Through Virtual Reality: Cross-Sectional Study
Source: JMIR Aging. 2026 Feb 25;9:e79224. doi: 10.2196/79224 (PMC12935412; doi:10.2196/79224)
Supplement: Multimedia Appendix 1 [file aging-v9-e79224-s001.docx]

**Neuropsychological Measures**

The MMSE assesses global cognition (1). Scoring ranges from 0 to 30 points, with higher scores indicating better cognitive performance. A corrected score below 22 suggests cognitive deterioration.

The Frontal Assessment Battery (FAB) (2) is a brief frontal functions battery with a maximum total score of 18 points. Higher scores indicate better frontal lobe functioning. The minimum score is 0. A cut-off score equal to or lower than 13.5 (Equivalent Score = 0) indicates frontal dysfunction.

The Rey-Osterrieth Complex Figure Test (ROCF) (3) evaluates visuospatial construction abilities and visual memory. It requires subjects to copy a complex geometric figure (ROCF-C) and subsequently reproduce it from memory at two intervals: immediately (30 seconds delay, ROCF-I) and after a delayed recall (20 minutes; ROCF-D). The minimum score is 0 and the maximum score is 36. The following cut-off scores indicate performance below the normal range: ROCF-C scores less than 23.76; ROCF-I scores less than 6.44; and ROCF-D scores less than 6.33.

The Prose Memory test (PM) (3) assesses verbal immediate episodic memory (after 30 seconds, PM-I) and delayed episodic memory (after a 20-minute delay, PM-D). Scoring ranges from 0 to 8 with a hierarchical approach. The cut-off values are: for PM-I, values less than 3.10; for PM-D, values less than 2.39.

The Trail Making Test (4) (TMT) assesses visual attention, processing speed, and mental flexibility. The score for each part is the time in seconds needed to complete the task, with no theoretical maximum score and 0 as minimum score. There is no fixed upper time limit, as scores are measured in seconds with lower scores indicating better performance. Cut-off scores corresponding to pathological performance are: 127 seconds or more for Part A; 294 seconds or more for Part B, 163 seconds or more for Score B minus A.

The Modified Five-Point Test (MFPT) (5) evaluates visuospatial flexibility. The main MFPT score is the total number of designs produced (MFPT-UD, maximum depends on individual’s speed). The minimum score is 0. Impairment is indicated by a MFPT-UD score of 10.05.

The verbal fluency test comprises three components (6): Phonemic Verbal Fluency (PF), Semantic Verbal Fluency (SF), and Alternate Phonemic/Semantic Fluency (AF). This test assesses verbal production, lexical abilities, and cognitive flexibility in switching between different subtasks. While there is no theoretical maximum score for these tests as they depend on the number of words generated. Pathological scores are: for PF below 17.77; for SF 28.34; and AF has a cut-off score of 12.7 words.

The Digit Forward (DF) test (7) assesses verbal short-term memory capacity. The Digit Span has a minimum score of 3 and a maximum score of 9. Scores below 4.26 are considered pathological.

The Corsi Forward (CF) test (7) evaluates visuo-spatial short-term memory. The Corsi block test has a minimum span score of 3 and a maximum score of 9. Scores below 3.46 are considered outside the normal range.

The Corsi-supra span (CSS) (8) evaluates visuospatial learning capabilities. The same sequence is repeatedly presented until the participant achieves the learning criterion (three consecutive correct reproductions) or reaches a maximum of 18 trials. The performance evaluation considers the subject’s baseline span capacity as measured by the CF. The minimum score is 0 and the maximum score is 29.16. The critical cut-off scores are 5, 7.5, and 10.5 with scores below these thresholds indicating impaired performance depending on CF baseline span.

The Tilburg Frailty Indicator (TFI) (9) is a brief self-report questionnaire that measures frailty in older adults across physical, psychological, and social domains. A score of 5 or higher typically indicates frailty. Scoring ranges from 0 to 15.

The Geriatric Depression Scale (GDS) short-form (10) is a brief self-report questionnaire that measures depressive symptoms. A score below 4 is considered normal. Scoring ranges from 0 to 15.

The Activities of Daily Living (ADL) (11) and Instrumental Activities of Daily Living (IADL) are assessment tools that measure functional independence in older adults. Scoring ranges from 0 to 6 for ADL and from 0 to 8 for IADL. Dementia should be suspected when both ADL is lower than 6 and IADL than 5. We also asked participants to self-report their informatic skills on PC on a five-point Likert scale ranging from insufficient to excellent.

**Object-Location Spatial Memory Task**

**Figure 1.** Virtual reality set-up with PC screen, PC keyboard, and foot-motion pad.


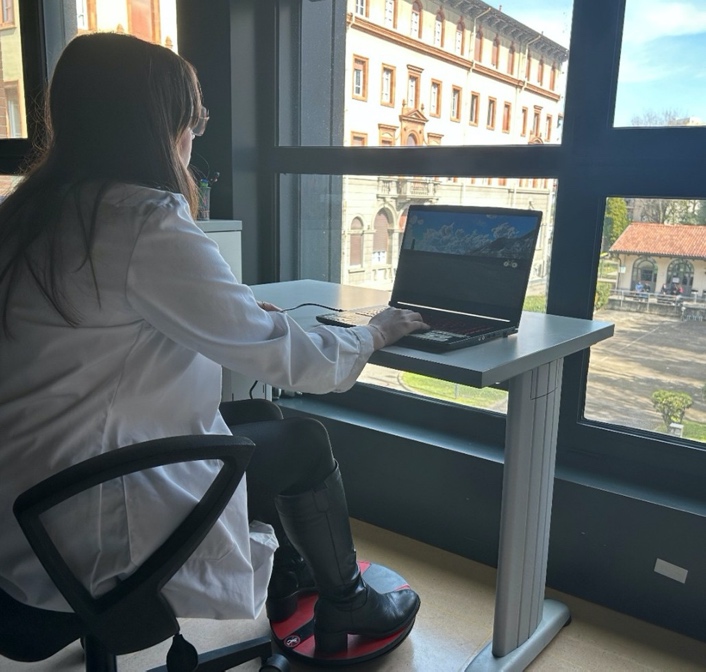


**Data Preparation and Statistical Analyses**

*Objective 1: Egocentric and Allocentric Spatial Performance*

The interquartile range method was applied to identify outliers within each group across the two spatial memory recall cue conditions for the dependent variable (DV; i.e., error) . Outliers were coded as missing values. We detected for the error DV 24/1280 outliers. Finally, skewness and kurtosis values guided the selection of the most suitable transformation to address violations of normality, with a square root transformation applied to the DV.

To investigate any differences in the two groups and recall cue conditions, we conducted linear mixed-effects ANOVA with restricted maximum likelihood according to Luke’s guidelines (13) with lme4 package. Post-hoc analyses with Bonferroni correction were carried out with emmeans package (14). To account for subject and object level variability, we added these terms to control them as random factors with random intercept (more complex models failed to converge). R formula for the models was: lmer(DV∼ spatial recall cue * group+covariate+(1|participant ID) + (1|object ID), control = lmerControl(optimizer = “bobyqa”), REML = T). Model assumptions were checked by visual inspection of the normality of residuals, collinearity, homogeneity of variance, and linearity plots. Crucially, linear mixed-effects is a great method that handles missing values properly without deleting the whole case (15). ANOVA effect size (f) was interpreted according to small = 0.1, medium = 0.25, and large = 0.4 (16).

The predictive power of the egocentric and allocentric performance on the DV to classify participants according to their group was investigated using a generalized linear logistic model (R formula: glm(group ~ egocentric+allocentric+covariate, family = binomial()). Odds ratio (OR) was used as a metric of association between the egocentric and allocentric score and the diagnosis (HC=0; aMCI=1).

Second, we investigated in the two groups the error performance across testing trials. To account for linear and non-linear trends, we computed a quadratic polynomial linear mixed-effect model. The R formula was: lmer(DV∼ spatial recall cue* group*poly(trial,2)+covariate+(1|participant ID) + (1|object ID), control = lmerControl(optimizer = “bobyqa”), REML = T).

Third, we computed an object-location binding measures as in Castegnaro and colleagues (17). First, we computed the mean error for each object and then we created a catchment area for each object with the radius as the half of the mean error. A binding error occurred if an object was recalled within the area of another object. Crucially, responses within any overlapping regions of adjacent objects were not counted as incorrect. Responses outside the areas were counted as invalid for this analysis. To examine the effect of group (aMCI vs. HC) and spatial recall cue (egocentric vs. allocentric) on binding errors, we implemented a Poisson mixed-effects model, appropriate for count data (R formula: glmer(formula = binding_errors ~ spatial recall cue*group+(1|participant ID)+(1|object ID), family = poisson). To account for inter participant and object variability we added these terms as random effects.

Lastly, to investigate the cognitive costs associated with switching between spatial frames of reference, we examined how performance differed when participants transitioned between egocentric and allocentric recall cues. We first identified each instance where a participant switched between reference frames during the recall trials. For each participant (grouped by ID), we created a lagged variable to track the previous trial’s reference frame (egocentric or allocentric). We categorized each trial into one of three recall cue switching conditions: transitions from allocentric to egocentric reference frames (‘allo🡪ego’), transitions from egocentric to allocentric reference frames (‘ego🡪allo’), or trials where no switch occurred (‘no switch’). Our primary outcome measure was the Euclidean distance error. To quantify switch-related performance effects, we calculated the mean distance error for each switch type condition within each participant (R formula: lmer(DV∼ switching condition *  group + covariate + (1|participant ID) , control = lmerControl(optimizer = “bobyqa”), REML = T). A logistic regression was used to explore which switching cue performance is a better predictor of aMCI diagnosis.

**Results**

Table 1 shows the detailed characteristics of the samples.

**Table 1**. Participants characteristics.

| Variable | aMCI, N = 40 | HC, N = 40 | p-value |
| --- | --- | --- | --- |
| Age (years) | 74 (6) | 72 (6) | 0.3 |
| Gender |  |  | 0.10 |
| F | 30 (75%) | 23 (57%) |  |
| M | 10 (25%) | 17 (42%) |  |
| Education (years) | 12.3 (4.5) | 13.9 (3.4) | 0.10 |
| MMSE (points) | 27.48 (2.10) | 28.52 (1.88) | 0.019 |
| FAB (points) | 14.99 (2.17) | 17.05 (1.12) | <0.001 |
| CF (points) | 4.73 (1.59) | 5.06 (1.00) | 0.5 |
| DF (points) | 5.69 (1.14) | 6.26 (0.95) | 0.022 |
| CSS (points) | 12 (7) | 19 (7) | <0.001 |
| TMT-A (sec.) | 36 (23) | 28 (14) | 0.3 |
| TMT-B (sec.) | 68 (60) | 43 (32) | 0.067 |
| TMT-BA (sec.) | 36 (40) | 18 (19) | 0.075 |
| ROCF-I (points) | 14 (7) | 20 (5) | <0.001 |
| ROCF-D (points) | 13.7 (6.3) | 19.4 (5.4) | <0.001 |
| PM-I (points) | 4.17 (2.27) | 5.82 (1.28) | <0.001 |
| PM-D (points) | 4.11 (2.22) | 5.84 (1.19) | <0.001 |
| ROCF-C (points) | 31.0 (5.1) | 33.4 (2.1) | 0.051 |
| MFPT (n° designs) | 20 (10) | 29 (7) | <0.001 |
| PF (n° words) | 32 (10) | 42 (8) | <0.001 |
| SF (n° words) | 45 (7) | 53 (9) | <0.001 |
| AF (n° words) | 26 (11) | 38 (10) | <0.001 |
| GDS (points) | 2.65 (2.62) | 1.93 (2.12) | 0.2 |
| ADL (points) | 5.95 (0.22) | 5.92 (0.27) | 0.7 |
| IADL (points) | 7.925 (0.474) | 7.925 (0.267) | 0.3 |
| TFI (points) | 4.35 (2.47) | 3.42 (2.34) | 0.082 |

aMCI: amnestic mild cognitive impairment; HC: healthy control: F: female; M: males; MMSE: mini-mental state examination; FAB: frontal assessment battery; CF: Corsi forward; DF: digit forward; CSS: Corsi-supra span; TMT-AB: trail making test part A, part B and part B minus A; ROF-I: Rey-Osterrieth figure immediate recall; ROF-D: Rey-Osterrieth figure delayed recall; ROF-C: Rey-Osterrieth figure copy; PM-I: prose memory immediate recall; PM-D: prose memory delayed recall; MFPT: modified five point test; PF: phonemic fluency, SP: semantic fluency; AF: alternate fluency; ADL: activities of daily living; IADL: instrumental activities of daily living; TFI: Tilburg frailty indicator. Welch two sample test was used to compare continuous variables in the two groups, and chi- squared to compare categorical variables in the groups. Mean and standard deviation are reported for continuous variables.

***Object-location memory binding in aMCI and HC and spatial memory recall cues***

**Figure 2.** Binding errors in the two groups.


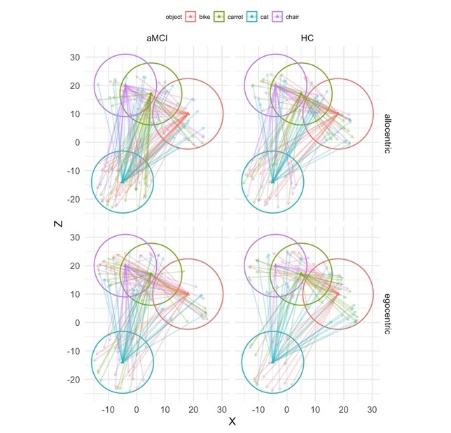


Only binding errors are reported. Arrows from encoding locations to objects’ areas show the object-location binding error displacement.

***Frames Switching Cost in aMCI and HC***

**Figure 3.** Unadjusted frame switching performance.


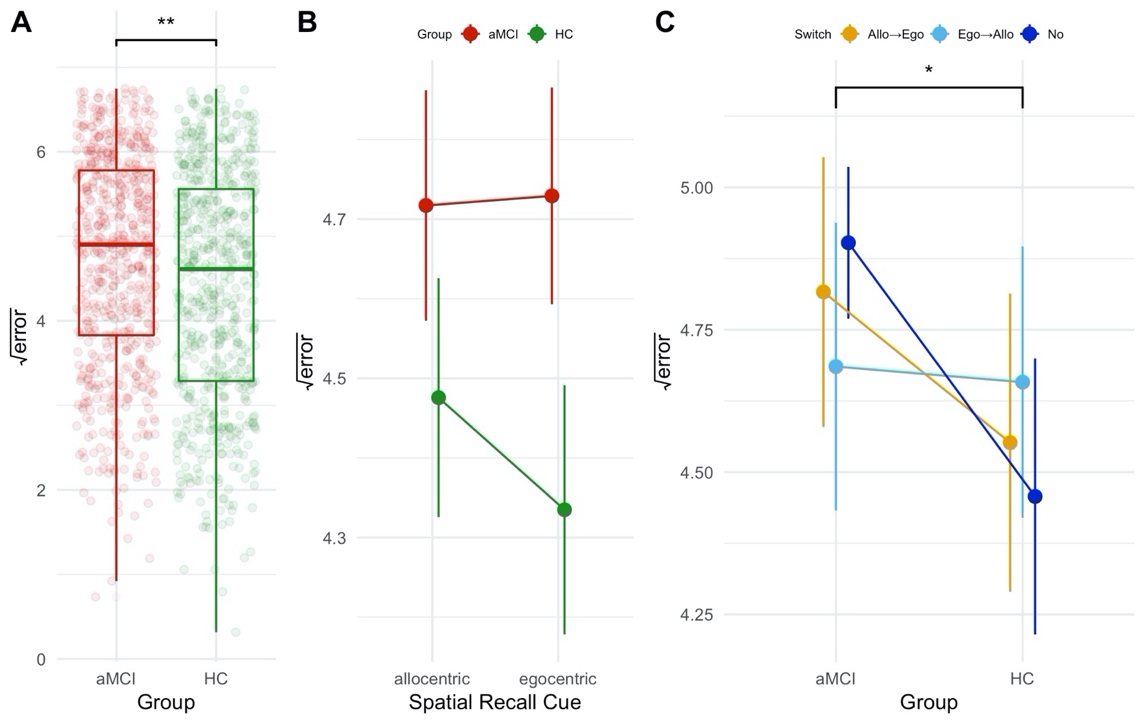


***Multiple Correlations***

The matrix in Table 2 shows performance in the whole sample (N = 80) between each neuropsychological test and egocentric and allocentric error. Crucially, egocentric error was significantly and negatively correlated with visuospatial long-term memory, visuospatial flexibility, and verbal set-shifting; allocentric error was significantly and negatively correlated only with global cognition.

**Table 2.** Multiple correlations between neuropsychological tests and spatial memory performance.

| Cognitive test | Correlation matrix | |
| --- | --- | --- |
|  | Allocentric  error | Egocentric  error |
| MMSE | -0.25* | -0.14 |
| CF | -0.19 | -0.16 |
| DF | -0.08 | -0.14 |
| CSS | -0.04 | -0.3** |
| TMT-A | 0.18 | 0.06 |
| TMT-B | 0.17 | 0.12 |
| TMT-BA | 0.15 | 0.15 |
| ROCF-C | 0.02 | -0.16 |
| ROCF-I | -0.18 | -0.14 |
| ROCF-D | -0.21 | -0.16 |
| PM-I | -0.01 | -0.08 |
| PM-D | -0.02 | -0.2 |
| FAB | -0.14 | -0.13 |
| MFPT | -0.04 | -0.35** |
| PF | -0.08 | -0.19 |
| SF | -0.15 | -0.18 |
| AF | -0.16 | **-0.41***** |

MMSE: mini-mental state examination; FAB: frontal assessment battery; CF: Corsi forward; DF: digit forward; CSS: Corsi-supra span; TMT-AB: trail making test part A, part B and part B minus A; ROCF-I: Rey-Osterrieth complex figure immediate recall; ROCF-D: Rey-Osterrieth complex figure delayed recall; ROCF-C: Rey-Osterrieth complex figure copy; PM-I: prose memory immediate recall; PM-D: prose memory delayed recall; MFPT: modified five point test; PF: phonemic fluency, SP: semantic fluency; AF: alternate fluency. *P<.05; **P<.01; ***P<.001.

**Explorative Analyses in aMCIsd and aMCImd**

***Spatial Memory Performance***

We used robust methods to compare encoding time and error difference and logistic regression between aMCI phenotypes given unequal group sizes.

A Wilcoxon rank-sum test was conducted to examine differences in encoding time between aMCImd and aMCIsd groups. Results revealed a significant difference in encoding time between the two groups (W = 60, p = 0.004). Participants in the aMCIsd group (M = 13.1, SD = 6.61) spent significantly more time in the encoding phase compared to participants in the aMCImd group (M = 7.48, SD = 3.74). This finding suggests that individuals with more severe domain impairments required substantially more time to learn spatial information during the task.

A robust mixed ANOVA using trimmed means was conducted to examine the effects of Phenotype (aMCImd vs. aMCIsd) and Spatial Recall Cue (egocentric vs. allocentric) on the spatial memory error. The analysis revealed no significant main effect of Spatial Recall Cue (0.863). Similarly, no significant main effect of Phenotype was observed (p = 0.374). The interaction between Spatial Recall Cue type and Phenotype was also non-significant (p = 0.720).

A robust logistic regression analysis was conducted to investigate whether allocentric and egocentric navigation performance could predict phenotype status. The model included allocentric and egocentric navigation measures as predictors. Results revealed that neither Allocentric error (p = .294) nor Egocentric error (p = .960) were significant predictors of classification status. The model intercept was also non-significant (p = .669). These findings suggest that neither allocentric nor egocentric navigation measures significantly discriminated between classification groups in this sample.

***Point Pattern Analysis and Spatial Recall Cue Biases***

We performed the recalled Cartesian coordinates analysis as outlined in the HC vs. aMCI section.

In the allocentric condition, the aMCIsd group exhibited 20% of points (n = 16) in the inner area and 80% (n = 64) in the outer area. Similarly, the aMCImd group showed 25% of points (n = 60) in the inner area and 75% (n = 180) in the outer area.

Binomial tests were conducted to compare point distributions with expected proportions based on area sizes (25% inner area, 75% outer area). For the aMCIsd group, the proportion of points in the inner area did not differ significantly from the expected distribution (binomial, p = 0.366). Similarly, the aMCImd group showed no significant deviation from the expected spatial distribution (binomial, p = 1).

Density calculations revealed that the aMCIsd group had a lower density in the inner area (0.03 points/unit^2^) compared to the outer area (0.04 points/unit^2^), yielding an inner-to-outer density ratio of 0.75. In contrast, the aMCImd group showed equal densities in both areas (0.12 points/unit^2^ for both inner and outer areas), resulting in a density ratio of 1.00. The difference in density ratios between groups was -0.25, indicating that aMCImd participants demonstrated a more balanced distribution of points between inner and outer areas compared to the aMCIsd group, which showed a preference for the outer area of the environment.

In the egocentric condition, the aMCIsd group showed 31.25% of points (n = 25) in the upper-left quadrant and 20.00% (n = 16) in the lower-right quadrant of a total of 80 data points. The aMCImd group displayed a similar pattern with 32.92% of points (n = 79) in the upper-left quadrant and 20.00% (n = 48) in the lower-right quadrant of a total of 240 data points.

Binomial tests were conducted to compare point frequencies between the two quadrants of interest within each group. For the aMCIsd group, the proportion of points in the upper-left quadrant to the lower-right quadrant did not differ significantly from chance (binomial, p = 0.211). In contrast, the aMCImd group showed a significant preference for the upper-left quadrant (binomial, p = 0.008).

Point density calculations (points per unit area) were performed for each quadrant. For the aMCIsd group, point density was 0.05 points/unit^2^ in the upper-left quadrant and 0.03 points/unit^2^ in the lower-right quadrant, yielding a density ratio of 1.56. For the aMCImd group, point density was 0.16 points/unit^2^ in the upper-left quadrant and 0.1 points/unit^2^ in the lower-right quadrant, resulting in a density ratio of 1.65.

The difference in density ratios between aMCIsd and aMCImd groups was -0.08, indicating similar spatial preferences across severity levels. Direct comparisons of quadrant densities between groups revealed differences in the upper-left (-0.11 points/unit^2^) and lower-right (-0.07 points/unit^2^) quadrants, with the aMCImd group showing higher densities in both quadrants.

Lastly, we performed the encoding path Cartesian coordinates analysis as outlined in the HC vs. aMCI section.

For the inner circle region, no significant difference was found between groups, with aMCIsd having 29.19% of encoding coordinates in the inner region compared to 29.15% for aMCImd patients (binomial, p = 0.917).

However, significant differences were observed in quadrant-specific movement patterns. The aMCIsd group showed more encoding coordinates in the bottom-right quadrant (16.38%) compared to the aMCImd group (14.18%; binomial, p < 0.001). Conversely, the aMCImd group showed more encoding coordinates in the top-left quadrant (25.21%) compared to the aMCIsd group (22.91%; binomial, p < 0.001). Figure 4 shows this section’s findings.

**Figure 4.** aMCIsd and aMCImd point pattern analysis.


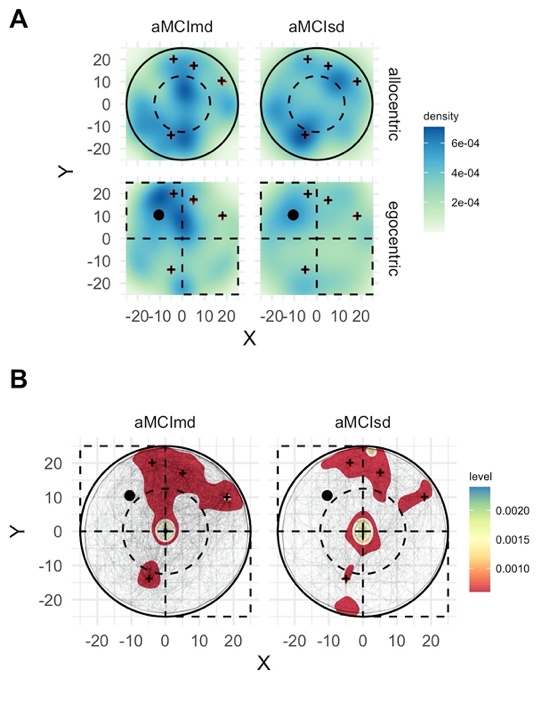


A) The plot shows the heat maps representing the density of response points, with dark blue colors indicating higher concentration. Black cross markers show encoding object positions. In the egocentric condition, the black dot represents the obelisk landmark (-10.5, 10.5), with dashed lines delineating the analyzed upper-left and lower-right quadrants; in the allocentric condition, the solid circle represents the arena boundary (radius = 25 units), with a dashed inner circle (radius = 12.5 units) separating central and annular regions.

B) The plot shows the heat maps representing the density of encoding path points, with dark blue colors indicating higher concentration. Black cross markers show encoding object positions. Black lines represent paths made by the participants during the encoding phase of the objects and their locations. Dashed lines delineate the analyzed upper-left and lower-right quadrants, whereas the solid circle represents the arena boundary (radius = 25 units), with a dashed inner circle (radius = 12.5 units) separating central and annular regions.

aMCIsd: amnestic mild cognitive impairment single domain; aMCImd: amnestic mild cognitive impairment multi-domain.

**References**

1. Magni E, Binetti G, Bianchetti A, Rozzini R, Trabucchi M. Mini-mental state examination: A normative study in Italian elderly population. Eur J Neurol. 1996;3(3):198–202.

2. Appollonio I, Leone M, Isella V, Piamarta F, Consoli T, Villa ML, et al. The Frontal Assessment Battery (FAB): normative values in an Italian population sample. Neurol Sci. 2005;26:108–116.

3. Carlesimo GA, Buccione I, Fadda L, Graceffa A, Mauri M, Lorusso S, et al. Stan­dardizzazione di due test di memoria per uso clinico. Breve racconto e figura di Rey. Nuova Riv di Neurol. 2002;12(1):1–13.

4. Siciliano M, Chiorri C, Battini V, Sant’Elia V, Altieri M, Trojano L, et al. Regression-based normative data and equivalent scores for Trail Making Test (TMT): an updated Italian normative study. Neurol Sci. 2019;40(3):469–77.

5. Battista P, Griseta C, Tortelli R, Guida P, Castellana F, Rivolta D, et al. The Modified Five-Point Test (MFPT): normative data for a sample of Italian elderly. Neurol Sci. 2021;42(6):2431–40.

6. Costa A, Bagoj E, Monaco M, Zabberoni S, De Rosa S, Papantonio AM, et al. Standardization and normative data obtained in the Italian population for a new verbal fluency instrument, the phonemic/semantic alternate fluency test. Neurol Sci. 2014;35(3):365–72.

7. Monaco M, Costa A, Caltagirone C, Carlesimo GA. Forward and backward span for verbal and visuo-spatial data : standardization and normative data from an Italian adult population. Neurol Sci. 2012;34(5):749–54.

8. Spinnler H, Tognoni G. Standardizzazione e taratura italiana di test neuropsicologici. Ital J Neurol Sci. 1987;8(Suppl):1–120.

9. Mulasso A, Roppolo M, Gobbens RJJ, Rabaglietti E. The Italian Version of the Tilburg Frailty Indicator: Analysis of Psychometric Properties. Res Aging. 2016 Nov;38(8):842–63.

10. Laudisio A, Antonelli Incalzi R, Gemma A, Marzetti E, Pozzi G, Padua L, et al. Definition of a Geriatric Depression Scale cutoff based upon quality of life: a population-based study. Int J Geriatr Psychiatry. 2018;33(1):e58–64.

11. Limongi F, Siviero P, Noale M, Gesmundo A, Crepaldi G, Maggi S. Prevalence and conversion to dementia of Mild Cognitive Impairment in an elderly Italian population. Aging Clin Exp Res. 2017;29(3):361–70.

12. Chersi F, Burgess N. The Cognitive Architecture of Spatial Navigation: Hippocampal and Striatal Contributions. Neuron [Internet]. 2015;88(1):64–77. Available from: http://dx.doi.org/10.1016/j.neuron.2015.09.021

13. Luke SG. Evaluating significance in linear mixed-effects models in R. Behav Res. 2017;49:1494–502.

14. Lenth RV. emmeans: Estimated Marginal Means, aka Least-Squares Means. 2018.

15. Brown VA. An Introduction to Linear Mixed-Effects Modeling in R. Adv Methods Pract Psychol Sci. 2021;4(1).

16. Cohen J. Statistical power analysis for the behavioral sciences. Routledge, editor. 2013.

17. Castegnaro A, Howett D, Li A, Harding E, Chan D, Burgess N, et al. Assessing mild cognitive impairment using object-location memory in immersive virtual environments. Hippocampus. 2022;32(9):660–78.

18. Lee SA, Miller JF, Watrous AJ, Sperling MR, Sharan A, Worrell GA, et al. Electrophysiological signatures of spatial boundaries in the human subiculum. J Neurosci. 2018;38(13):3265–72.
